# Toxicity of nitrophenolic pollutant 4-nitroguaiacol to terrestrial plants and comparison with its non-nitro analogue guaiacol (2-methoxyphenol)

**DOI:** 10.1038/s41598-024-52610-6

**Published:** 2024-01-25

**Authors:** Maksimiljan Adamek, Anja Kavčič, Marta Debeljak, Martin Šala, Jože Grdadolnik, Katarina Vogel-Mikuš, Ana Kroflič

**Affiliations:** 1 Department of Analytical Chemistry, National Institute of Chemistry, Hajdrihova 19, 1000 Ljubljana, Slovenia; 2 Department of Molecular Biology and Nanobiotechnology, National Institute of Chemistry, Hajdrihova 19, 1000 Ljubljana, Slovenia; 3 Biotechnical Faculty, Department of Biology, University of Ljubljana, Jamnikarjeva 101, 1000 Ljubljana, Slovenia; 4 Theory Department, National Institute of Chemistry, Hajdrihova 19, 1000 Ljubljana, Slovenia; 5 Jozef Stefan Institute, Jamova 39, 1000 Ljubljana, Slovenia; 6 Department of Catalysis and Chemical Reaction Engineering, National Institute of Chemistry, Hajdrihova 19, 1000 Ljubljana, Slovenia

**Keywords:** Ecology, Environmental sciences

## Abstract

Phenols, and especially their nitrated analogues, are ubiquitous pollutants and known carcinogens which have already been linked to forest decline. Although nitrophenols have been widely recognized as harmful to different aquatic and terrestrial organisms, we could not find any literature assessing their toxicity to terrestrial plants. Maize (monocot) and sunflower (dicot) were exposed to phenolic pollutants, guaiacol (GUA) and 4-nitroguaiacol (4NG), through a hydroponics system under controlled conditions in a growth chamber. Their acute physiological response was studied during a two-week root exposure to different concentrations of xenobiotics (0.1, 1.0, and 10 mM). The exposure visibly affected plant growth and the effect increased with increasing xenobiotic concentration. In general, 4NG affected plants more than GUA. Moreover, sunflower exhibited an adaptive response, especially to low and moderate GUA concentrations. The integrity of both plant species deteriorated during the exposure: biomass and photochemical pigment content were significantly reduced, which reflected in the poorer photochemical efficiency of photosystem II. Our results imply that 4NG is taken up by sunflower plants, where it could enter a lignin biosynthesis pathway.

## Introduction

Methoxyphenols are the main component of the most abundant natural aromatic polymer lignin. They are released into the atmosphere during biomass combustion, which is a large source of atmospheric trace gases and particulates^1^. Biomass burning for residential heating is expected to become the major source of primary particulate matter emissions over the next decade^2^. Methoxyphenol emissions depend on fuel type and have been reported in the range from as little as 1 mg kg^−1^ to as much as 10,000 mg kg^–1^ fuel burnt^3–5^.

Biomass burning emissions are toxic to organisms^6^ and their toxicity can be further enhanced by atmospheric transformation into nitroaromatic compounds^7^. Nitroaromatic compounds have been extensively studied in the past due to their environmental contamination, stability, resistance to degradation, and toxicity and mutagenicity^8^. In living organisms, nitroaromatic compounds and their degradation products cause oxidative stress, covalently bind to proteins or DNA, affect enzyme activity, and exert mutagenic and genotoxic effects^8–10^. Very recently, disintegration of an eukaryotic cell membrane bilayer by phospholipid extraction has been observed after exposure to a mixture of nitrophenols, which was further linked to lung cell damage and increased penetration of airborne pollutants, causing various diseases^11^. Different nitroaromatic compounds have already been determined in atmospheric samples^6^, snow samples^12^, and soil samples^13^.

Due to atmospheric deposition by precipitation and potential accumulation in the soil, nitroaromatic compounds inevitably come into contact with plant root systems. They have been shown to accumulate in plant roots, leaves, and stems^14,15^. Phytotoxicity of the well-studied nitroaromatic pollutant 2,4,6-trinitrotoluene and its derivatives has already been established^16,17^, whereas nitrophenols have only vaguely been connected to forest decline^18,19^.

To date, very little attention has been given to nitrated derivatives of methoxyphenols. Nitrated phenols can be directly emitted in biomass burning plumes^20^. Moreover, major wood-derived methoxyphenol guaiacol (GUA, 2-methoxyphenol) can be also transformed into its nitrated derivatives 4-nitroguaiacol (4NG), 5-nitroguaiacol (5NG), 6-nitroguaiacol (6NG), and 4,6-dinitroguaiacol (4,6DNG) due to reactions with atmospheric reactive nitrogen species^21–25^. If nitration indeed increases toxic potential of aromatic compounds, the toxicity of biomass burning atmospheric aerosols may increase with their residence time in the atmosphere^26^. Acute toxicity of GUA, 4NG, 6NG, and 4,6DNG has recently been studied for *Vibrio fisheri*^27^. However, the effects of methoxyphenols and their nitroaromatic derivatives on plants have not yet been established. Due to the possibly large emissions during biomass burning, apparent toxicity, and the inevitable contact of these compounds with plants, methoxyphenols and their nitroaromatic derivatives could, among others, exert serious negative effects on plant growth, health, and productivity, resulting in negative economic and societal consequences.

In this paper, we present the first toxicity assessment of GUA and 4NG to terrestrial plants. Two species were selected based on their simple cultivation: maize (*Zea mays* L.) as a representative monocotyledonous species and sunflower (*Helianthus annuus* L.) as a representative dicotyledonous species. The acute physiological response of plants to xenobiotic exposure through a hydroponics system was evaluated under controlled conditions in a growth chamber. The effect on biomass, photochemical efficiency of photosystem II and photosynthetic pigment content, and the uptake of the investigated xenobiotics by plants was investigated.

## Results and discussion

Our experimental set-up consisted of groups of maize and sunflower plants typically treated for two weeks in beakers with diluted Hoagland’s nutrient solution with and without added xenobiotics. The two investigated compounds were GUA and 4NG. The concentrations used (0.1, 1.0, and 10 mM) were chosen in line with common toxicological practice to efficiently cover a wide range of concentrations and were in the same range as those from the study on the effects of nitrobenzenes on plants^10^, whereas the highest (10 mM) concentration was not tested for 4NG exposure due to solubility limitations.

A simple visual inspection of treated plants showed that the added organic compounds significantly affected plant growth. Visually, the difference was more prominent in the case of 4NG for 0.1 mM and 1.0 mM concentrations, while 10 mM GUA effectively killed all plants in a beaker already after one week of exposure. Therefore, plants exposed to 10 mM GUA are not included in the following analysis; they were too damaged during cultivation.

**Figure 1:**
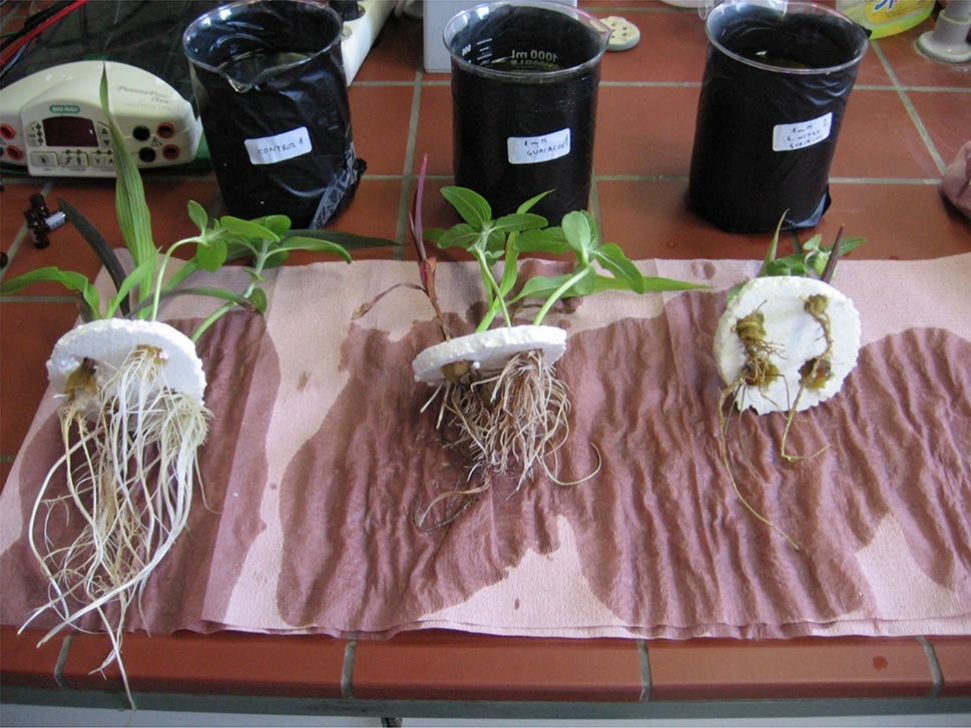
Visual assessment of plants. Plants (sunflower and maize) after two weeks of cultivation in hydroponics without and with the addition of xenobiotics. From left to right—control, 1 mM guaiacol (GUA) and 4-nitroguaiacol (4NG).

In the case of sunflower exposure to GUA, a more branched root system was developed, which started browning at a higher (1.0 mM) concentration (Fig. 1, middle). The reddish color can stem from peroxidase enzymatic activity, which is known to promote red tetraguaiacol formation^28^. Very recently, a phenol-coupling mechanism has also been proposed for the cytochrome P450 family of enzymes, which could also result in colored conjugates containing multiple aromatic rings^29^. The roots of maize were more damaged compared to sunflower exposure to GUA and red coloration was evident already at 0.1 mM exposure (data not shown). In the case of 4NG, however, roots of both plant species were fairly destroyed at both concentrations and red color was not observed. But this could also be due to substantial rotting. These visual observations will be considered with other complementary data supporting given observations.

### Biomass

**Figure 2:**
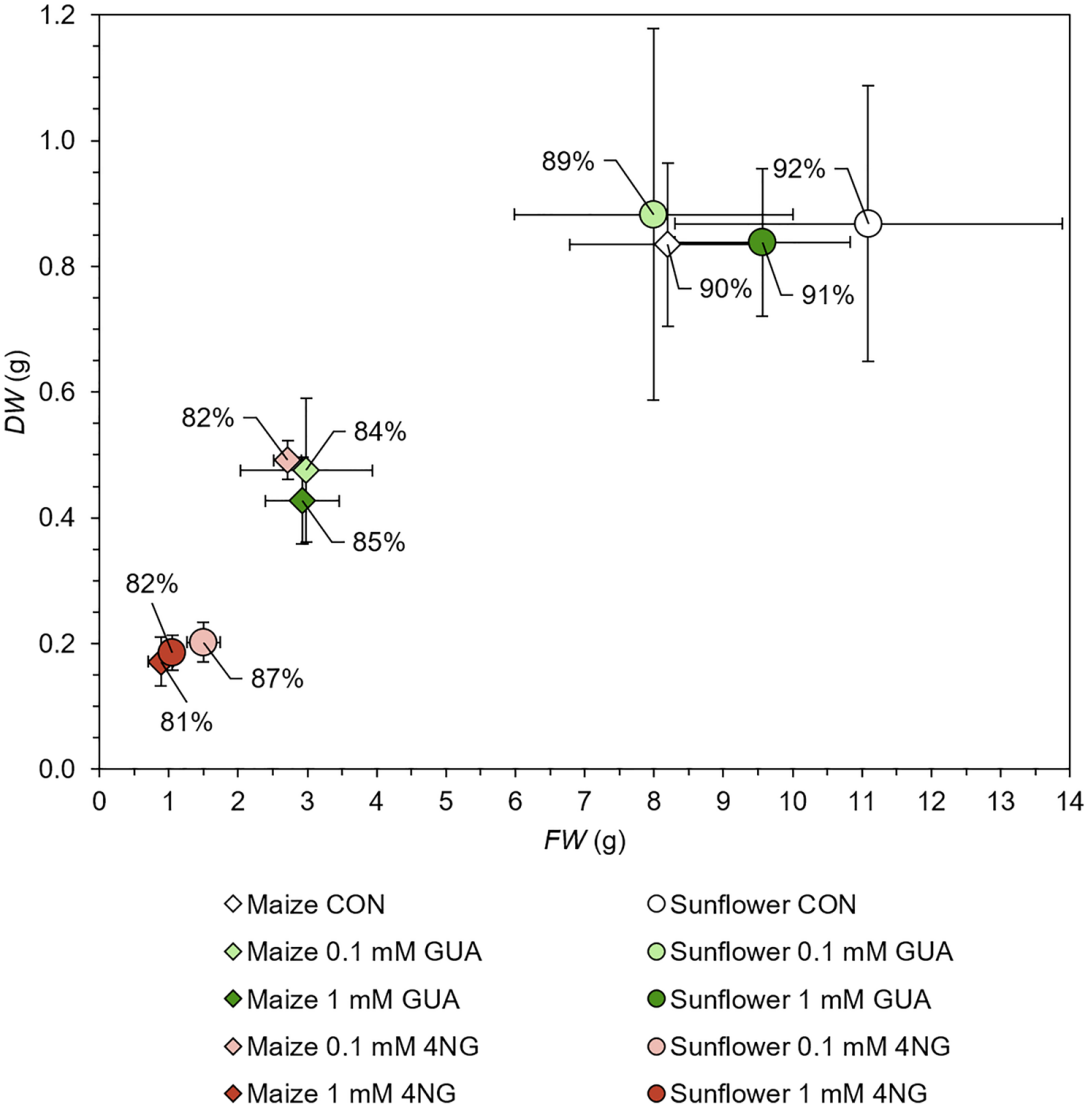
Biomass. Dry weight (DW) vs. fresh weight (FW) of maize and sunflower after two weeks of exposure to 0.1, and 1.0 mM GUA and 0.1, and 1.0 mM 4NG. Control plants (CON) are shown for comparison. Points represent mean values of DW and FW for each exposure (n = 4), error bars are standard errors (SE). The percentage number next to each data point corresponds to the mean water content of the plants.

A simple visual assessment of exposed plants is confirmed by comparing their biomass with control samples. Fresh (FW) and dry (DW) roots and shoots weights were determined and statistically evaluated separately in Figure S1. In Fig. 2, however, root and shoot masses are considered together for easier presentation.

In the case of maize, all exposures significantly suppressed plant growth, which can be connected with the damage of the root system. The major effect was observed for 1.0 mM 4NG, where no root biomass was available due to the rotting of the roots. Compared to the control plants, water content was reduced for all treated maize plants (WC; 81–85% in comparison to 90% in control plants), in line with the root system damage, aggravating water and mineral nutrient uptake. On the other hand, only 4NG exposure considerably affected sunflower biomass, which can be again linked with the root damage. Moreover, sunflower WC remained very high (> 87%, except for 1 mM 4NG exposure 82%), which points to its better adaptation to the exerted stress compared to maize.

Statistical analysis of separate DW and FW of roots and shoots in SI Figure S1, however, only partly supports above observations. Root weights of 1.0 mM 4NG exposure are not shown, because the roots of both corresponding plants were almost completely destroyed. In maize, we found no statistically significant effect on root weight for all other exposures, although visually, the root system changed considerably. Despite the statistically significantly reduced FW and DW of 1.0 mM 4NG treated maize shoots compared to control plants, no statistically significant difference was observed between the different treatments. In contrast to maize, no statistically significant differences were observed for the sunflower FW and DW compared to the control plants and between the different treatments. Insignificant differences are probably due to large biological variability and small sample sizes used in this screening study.

We can conclude that sunflower copes better with GUA-based xenobiotic exposure than maize. The reason is probably the difference in plant cell wall composition and phenolic compounds metabolism between dicots and monocots^30,31^, which warrants further investigation.

### Photochemical efficiency and photosynthetic pigments

Nitroguaiacols are classified as respiratory uncouplers of oxidative phosphorylation^27^; therefore, the effect of 4NG on plant respiration is expected. GUA, on the other hand, belongs to Class I toxicants—nonpolar narcosis, which is typical of nonreactive chemicals with baseline toxicity. Although no direct effect on photosynthesis is expected based on this parametrization, we could observe some significant differences between the treated and control plants.

Maximal photochemical efficiency (*Fv/Fm*) at the beginning of the experiment and after one and two-weeks cultivation is shown in SI Figure S2, including a detailed statistical analysis. Statistically significant reduction in photochemical efficiency was observed after two-weeks exposure to 1.0 mM 4NG for both plant species. In the case of GUA, photochemical efficiency was significantly reduced only for the 10 mM exposure, which is consistent with the collapsed integrity after one-week cultivation. Note that sunflower plants showed quite some variability already at the beginning of the experiment (*t* = 0).

On the other hand, at the end of experiment, the concentration of photosynthetic pigments in plant tissues was significantly reduced for all pigments and exposures in the case of maize (Figure S3). Chlorophyll *a* and carotenoids content tend to be lower after exposure to 4NG compared to GUA. The effect of GUA on the photosynthetic pigment concentration in sunflower plants was inconclusive due to the large variability but was considerably smaller than the effect of 4NG. In the case of 4NG exposure, the reduction of photosynthetic pigment concentration was statistically significant compared to control plants.

**Figure 3:**
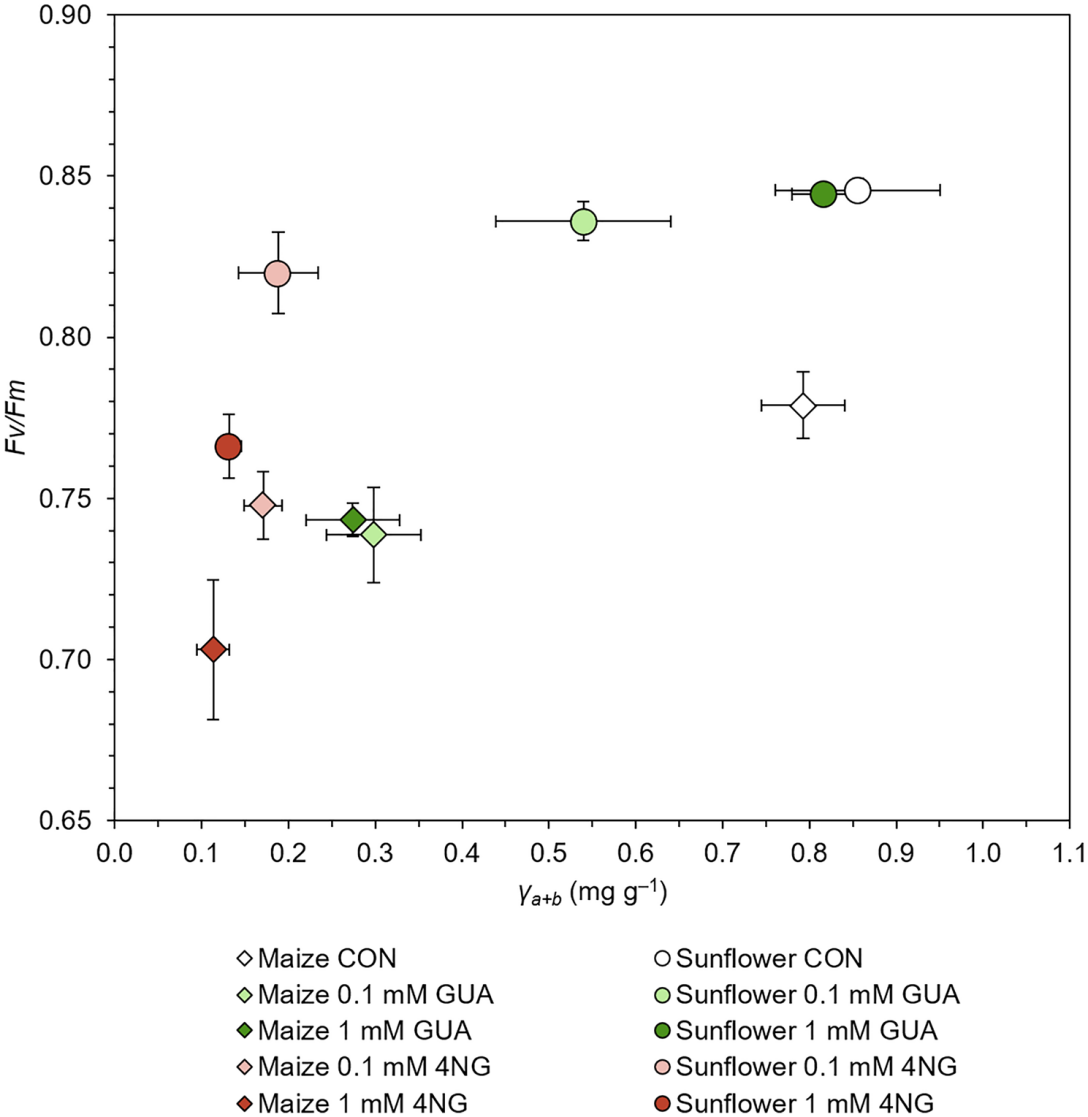
Photosynthesis. Maximal photochemical efficiency (*Fv/Fm*) after two-week exposure of maize and sunflower to 0.1, and 1.0 mM guaiacol (GUA) and 0.1, and 1.0 mM 4-nitroguaiacol (4NG) vs. the sum of photosynthetic pigments (*Y*_a+b_; sum of Chlorophylls *a* and *b* expressed in mg of photosynthetic pigments per g of dry plant biomass) after two weeks of exposure. Control plants (CON) are shown for comparison. Data points represent mean values of *Fv/Fm* (n = 8) and a sum of photosynthetic pigments (n = 4), error bars are standard errors (SE).

These observations can be connected with impaired metabolism and/or integrity of treated plants, which is reflected in reduced amount of photosynthetic pigments and photosynthetic efficiency, and is evident from the reduced biomass presented in Figure S1. We further compare *Fv/Fm* after two weeks of exposure with the concentration of photosynthetic pigments (i.e., sum of Chlorophylls *a* and *b*) in sunflower and maize at the end of the experiment (Fig. 3). For both plant species, photosynthetic pigment concentration reduction due to xenobiotic exposure generally coincided with lower photochemical efficiency of exposed plants compared to control plants (the lower the pigment content, the lower the *Fv/Fm*). In both cases, 4NG affected photochemical efficiency of plants more than GUA compared to control plants. In the case of sunflower, considerable reduction was only observed for 4NG exposures, which agrees with the retained integrity of sunflower plants upon low and medium GUA exposure.

Similar diagrams for different photosynthetic pigments are shown in Figure S4. In maize, significantly lower Chlorophyll *a* concentration at the end of the experiment coincided with *Fv/Fm* lowering after two weeks of exposure. Although lower Chlorophyll *a* concentration was measured in 4NG exposed maize plants, they showed a comparable gross *Fv/Fm* to the GUA exposure. This was similar for Chlorophyll *b* and carotenoids. In sunflower, the concentration of all three investigated photosynthetic pigments was much less affected by GUA exposure (for both concentrations), whereas the decrease in photosynthetic pigments after a two-week exposure to 4NG was again accompanied by a considerably lower *Fv/Fm* ratio. The effects of xenobiotics on the photosynthesis of both tested plant species could be mainly attributed to the damage of the root system, which consequentially affected mineral nutrient uptake, essential for the normal synthesis of photosynthetic pigments and functioning of photosynthetic apparatus.

### Clustering analysis

**Figure 4:**
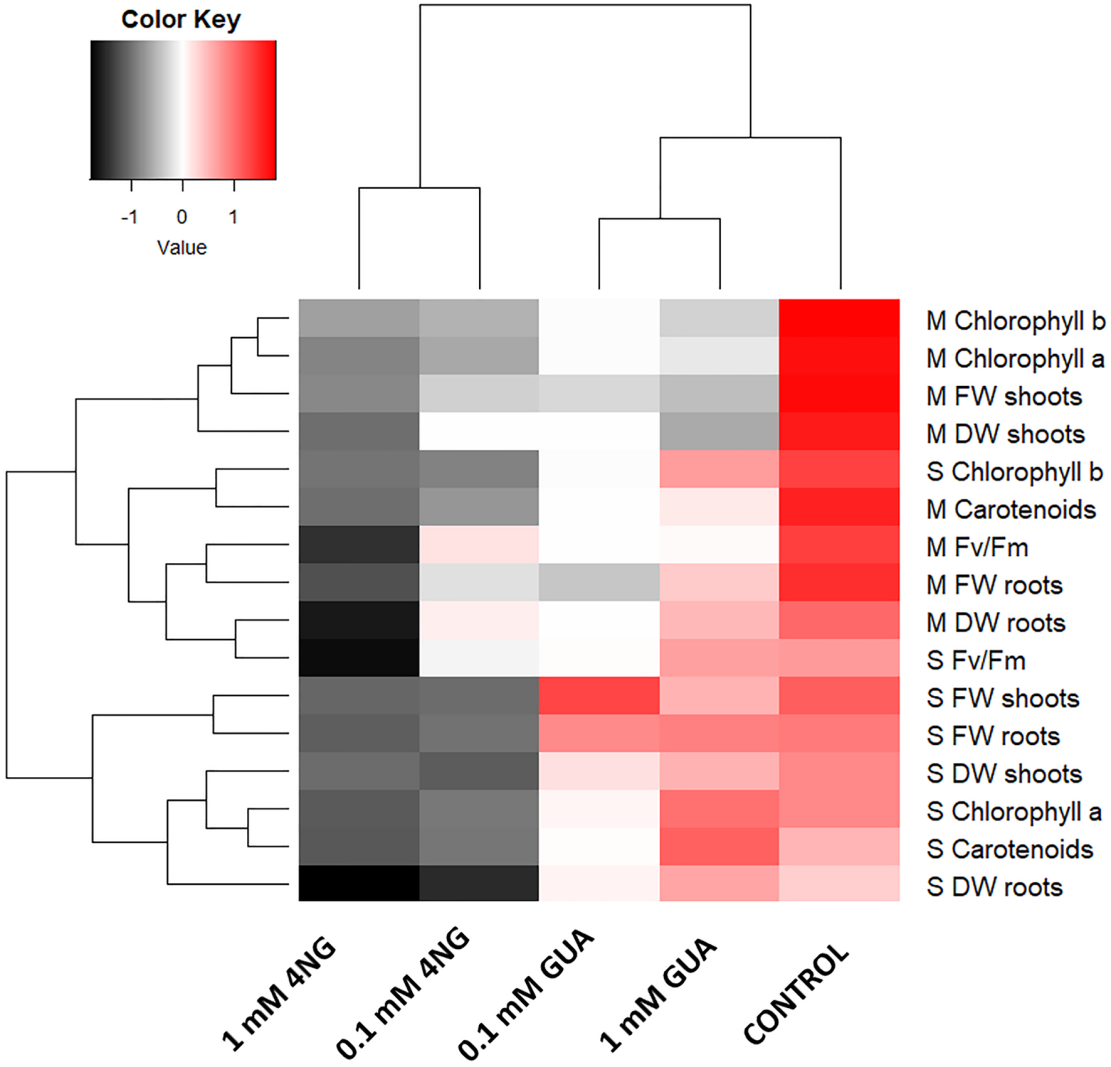
Clustering analysis. Two-dimensional hierarchical clustering based on Euclidian distances of z-transformed averages of maize (M) and sunflower (S) traits at different exposures (0.1, and 1.0 mM GUA and 0.1, and 1.0 mM 4NG). Control plants (CON) are added to the analysis for comparison; fresh weight (FW), dry weight (DW), and maximal photosynthetic efficiency (*Fv/Fm*) after two weeks of exposure.

The result of two-dimensional clustering analysis based on Euclidian distances is shown in Fig. 4.

The obtained heat chart nicely demonstrates and confirms a greater effect of 4NG exposure on plant physiology compared to GUA exposure, which clusters closer to the control plants. Moreover, a difference between maize and sunflower responses forming two separate clusters is also evident, with sunflower being more tolerant to GUA, which has already been discussed. In the case of maize, 1.0 mM 4NG deviates substantially from all the other exposures (0.1 and 1.0 mM GUA, and 0.1 mM 4NG).

### Xenobiotic uptake

To assess whether or not GUA and 4NG remained in nutrient solutions after the exposure or were taken up by plants during the treatment, exposure solutions were first analyzed by HPLC for the presence of organics after one week of exposure. Chemical analyses revealed that after one week of plant cultivation, there was no GUA left in the beakers for the lower two GUA concentrations (0.1 and 1.0 mM), while roughly 3.5 mM residual GUA was determined in 10 mM exposure samples (note the plants had already been completely destroyed by then). In the second week, we monitored GUA concentrations more regularly and, surprisingly, found no GUA in the samples already one day after nutrient solutions containing xenobiotic had been exchanged (day 8, approx. 24 h exposure). Blank experiments (without plants) ruled out GUA adsorption to the beaker walls and its evaporation from the solution, so GUA must have been taken up by plants or was adsorbed on the roots. Although we could speculate about GUA absorption into the root tissue in the case of sunflower, where GUA was presumably metabolized to red-colored products (see above), we can neither confirm nor exclude GUA uptake by maize, because maize and sunflower plants were cultivated together in every beaker and we cannot distinguish between them on the basis of this analysis.

**Figure 5:**
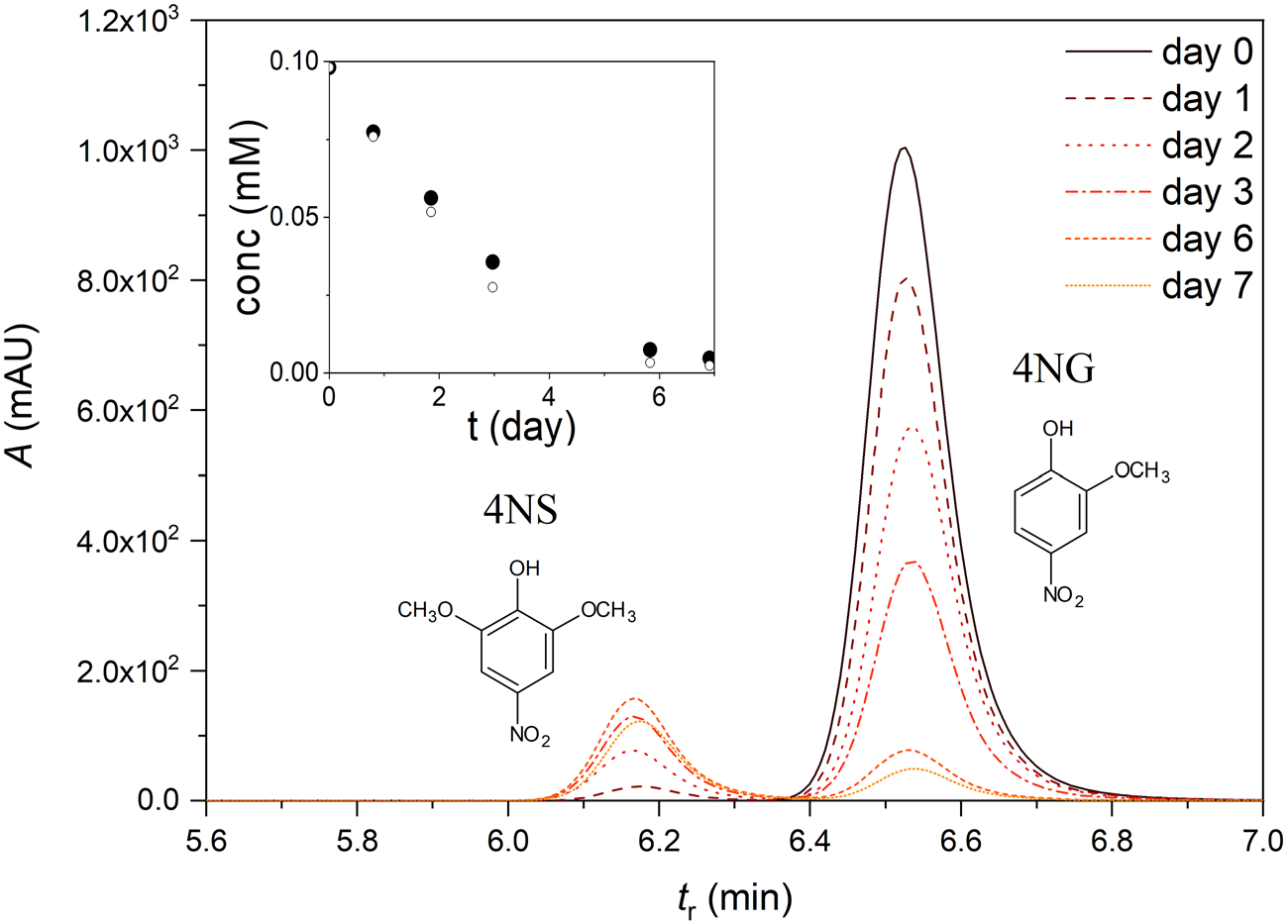
Exposure solution. HPLC chromatograms of decreasing 4-nitroguaiacol (4NG) concentrations during a week-long exposure. The peaks at approximately 6.5 min correspond to 4NG, and the appearing peaks at approximately 6.2 min were later confirmed as 4-nitrosyringol (4NS). Inset: 4NG decay in the exposure solution obtained from time-dependent chromatograms; the different symbols denote two parallel experiments.

On the other hand, 4NG concentration in the beakers was decreasing during the experiment, but never dropped to zero, which is shown in the inset in Fig. 5 for the 0.1 mM exposure. Adsorption to the walls and evaporation were again excluded by blank experiments. Furthermore, another peak appeared in the chromatograms of exposure solutions next to the 4NG peak (Fig. 5), which implies 4NG was also taken up and metabolized by at least one of the cultivating plants.

Another set of experiments was thus conducted to investigate the uptake of the studied compounds by treated plants. The presence of GUA and 4NG in root samples was first investigated by ATR-FTIR. Infrared spectra of control and treated plants are shown in Figure S5. As expected, the magnitude and the frequency of the difference spectra within controls, and between controls and treated samples differed significantly. The main differences can be attributed to the degradation of the roots due to GUA and 4NG exposure. However, in addition to these spectral changes, some significant band overlaps were also observed between the difference spectra of the treated samples and pure GUA and 4NG solutions^32^. These band overlaps served as the first evidence of the uptake of GUA and 4NG in maize and sunflower roots.

**Figure 6:**
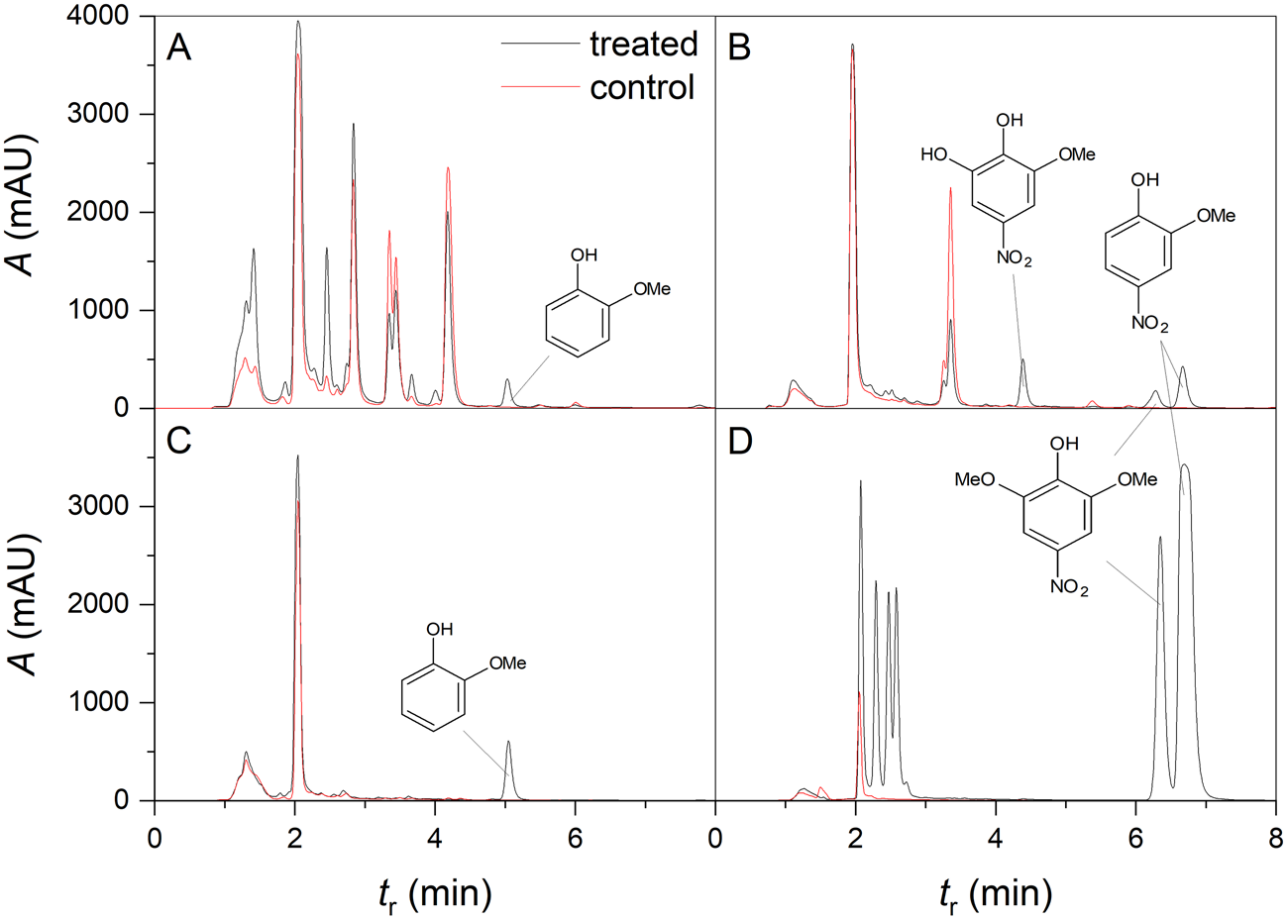
Root extracts. HPLC chromatograms of A,B) maize and C,D) sunflower root extracts for plant exposure to A,C) 0.1 mM guaiacol (GUA) and B,D) 0.1 mM 4-nitroguaiacol (4NG) compared to control samples. The peaks corresponding to nitroaromatic metabolites were assigned as 3-methoxy-5-nitrocatechol and 4-nitrosyringol (4NS).

Further chromatographic analyses of the corresponding root extracts confirmed that GUA was present in all GUA exposed samples. Besides 4NG, two other nitrated aromatic metabolites were also detected in the case of 4NG exposure (Fig. 6), one of them corresponding to the unknown peak found in the exposure solutions. Finally, the identity of this peak, which we found in the extracts of both plant species, was confirmed as 4-nitrosyringol (4NS) by LC–MS/MS and comparison with the authentic standard. The other metabolite, which was only detected in exposed maize lyophilizates, was tentatively assigned as 3-methoxy-5-nitrocatechol based on LC–MS/MS analysis. It is known that detoxification often starts with hydroxylation inside the cell cytoplasm and in the next step, water-soluble conjugates are usually formed that are further processed and stored in a vacuole^33^. We, however, detected a methoxylated product (i.e., 4NS), which is typical of lignin biosynthesis pathways and could imply the start of 4NG transformation in a plant cell wall^34^.

Despite more than one scientific evidence that points to the uptake of GUA and 4NG by the treated plants, xenobiotic absorption into the plant tissues is still not unambiguously confirmed. Theoretically, three different scenarios can explain the presence of GUA, and 4NG and its metabolites in root samples: (i) strong xenobiotic adsorption on the root surface that prevented washing GUA and 4NG away by rinsing and resulted in their detection in the powdered root lyophilizate and corresponding extracts, (ii) bacteria from the plants themselves caused rhizodegradation of dissolved 4NG in a beaker due to microbial activity evolved within the root system, or (iii) 4NG was taken up by plants and phytodegraded by entering plant metabolic pathways, which was followed by (selective) excretion of plant metabolites by the roots. Although the presence of symbiotic bacteria due to the restricted algae growth in a hydroponics system is very unlikely, it is also not clear whether GUA and 4NG are only present in plant root tissue or systemically distributed over the whole plant.

**Figure 7:**
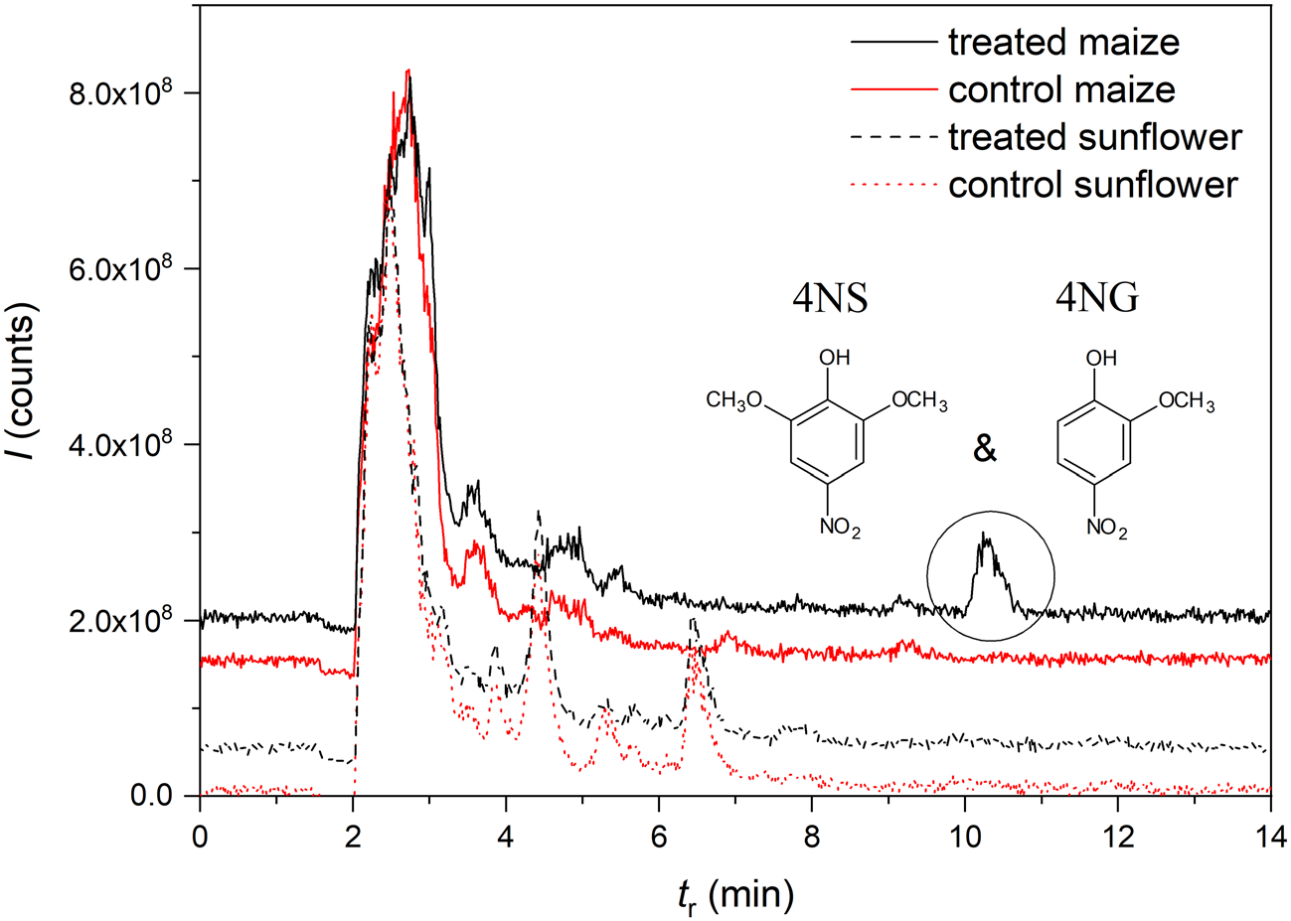
Xenobiotic uptake. Typical total ion chromatograms (TIC) of maize (dashed lines) and sunflower (solid lines) shoot extracts for plant exposure to 0.1 mM 4NG (black) compared to control samples (red). The peak at approximately 10 min confirms xenobiotic uptake by plants and includes fragment ions of both, 4-nitroguaiacol (4NG) and 4-nitrosyringol (4NS).

Therefore, lyophilized shoots, which were never in direct contact with exposure solutions, were also extracted and analyzed for trace nitrated phenols by LC–MS. Note that GUA does not ionize in ESI and could not be detected by this experiment. We finally determined 4NG and 4NS in the roots as well as in the upper parts of 4NG-exposed sunflower plants (Fig. 7 and Figure S6), which unequivocally confirmed the uptake of 4NG from the solution and its transformation by plant metabolism. This further implies that systemic effects of toxic nitrophenols can be anticipated and that the observed physiological response cannot be only attributed to the damage caused to the roots during the exposure. In maize shoots, however, metabolites of xenobiotics were not detected.

In summary, selected wood-lignin-related-phenolic pollutants, GUA and its nitrated derivative 4NG, affected physiology of maize (as a representative of monocotyledonous plants) and sunflower (as a representative of monocotyledonous dicotyledonous) plants negatively, 4NG more than GUA, in line with recent reports for aquatic organisms^27^. Both plant species exhibited root damage, photosynthetic pigment reduction, poor photochemical efficiency, and overall decrease in biomass; however, sunflower appeared to be less stressed than maize. The amount of chlorophyll content was directly related to the photochemical efficiency of photosystem II; the lower the pigment concentration the lower the photosynthetic efficiency.

In sunflower, a pronounced branched root system was observed accompanied by browning at medium concentrations and deterioration at the highest GUA concentration. Root browning can be connected with enzymatic activity and potential formation of red tetraguaiacol in GUA-exposed roots, which should be investigated further. At low and medium GUA concentration, only minor biomass, WC, and photochemical efficiency loss were found in sunflower, indicating physiological plasticity for this plant species. Our results further imply that 4NG is taken up by sunflower plants where it may have entered the lignin biosynthesis pathway and exerted systemic effects beyond the physiological response caused by the damaged roots. This, however, warrants further investigation. Maize, on the other hand, responded negatively already to the lowest GUA concentrations and we could not confirm 4NG uptake into the plants. However, an additional 4NG analogue, 3-methoxy-5-nitrocatechol, was found in maize root extracts, which could not be detected in sunflower samples, indicating possible transformation of 4NG in maize.

Expectedly, physiological responses observed for sunflower and maize depended on the concentration of xenobiotic tested. The highest concentrations used turned out to be lethal for both plant species. Phenolic compounds, including GUA, have been detected in rivers, drinking water, suspended particulate matter, and sediment fractions with concentrations ranging between ng L^–1^ and μg L^–1^^35–38^. Although environmental concentrations of these two mobile chemicals are well below the lowest concentration used in this study (0.1 mM or 12.4 mg L^–1^ GUA), pollutants accumulation in soil is expected at least to some extent. Moreover, acute toxicity assessment is usually considered at higher concentrations than relevant for the chronic exposure, which is out of the scope of this study. Future work on this topic is needed for an in-depth ecotoxicity assessment, including a larger number of physiologically relevant parameters (e.g., also enzyme activities), effects of other nitrophenolic compounds and their synergistic effects, and—considering these are predominantly airborne pollutants—foliar exposure.

## Materials and methods

### Experimental design

The effects of GUA and 4NG were studied on hydroponically grown maize and sunflower in two independent experiments. Seeds (obtained from Semenarna Ljubljana) were sterilized using 10% sodium hypochlorite^39^ and germinated on vermiculite (Agra-vermiculite, RHP, the Netherlands) that was regularly watered with Hoagland’s nutrient solution^40^. Two weeks old seedlings were transferred to a hydroponics system consisting of 1 L glass beakers covered with black plastic bags (to prevent algae growth and xenobiotic photodegradation) and filled with 4-times diluted Hoagland’s nutrient solution. Two maize and two sunflower seedlings were cultivated in each beaker (24 maize and 24 sunflower seedlings altogether) in a growth chamber with a 16/8 h day/night photoperiod, cool white fluorescent illumination of 550 μmolm^−2^ s^−1^, at a constant temperature of 20 °C and 50% humidity. Nutrient solution was continuously aerated. Fresh Hoagland’s nutrient solution was regularly added to keep the beaker full and maintain mineral nutrition.

After one week, the solution in a beaker was exchanged for a fresh 1 L nutrient solution which contained 0 (control solution) or either 0.1, 1.0, or 10 mM GUA or 0.1, or 1.0 mM 4NG (exposure solutions). Beakers with xenobiotics but without plants were also incubated to assess the potential evaporation of relatively volatile xenobiotics. Two beakers per treatment were used, whereby each beaker contained two maize and two sunflower plants, yielding four specimens for every treatment altogether. The volume of nutrient solution in the beakers was again maintained by adding fresh Hoagland’s solution (control) or exposure solutions (as required) and all solutions were exchanged after one week of cultivation. The plants were allowed to grow for another week, resulting in two-week treatment altogether (except at 10 mM GUA, see Results and discussion section). The concentration of GUA and 4NG in the exposure solutions was measured immediately after the transfer of seedlings to the exposure solutions (within an hour), after one week (before exchanging the nutrient solution for a fresh one) and then 8, 11, 12, and 13 days after the beginning of the exposure by high-performance liquid chromatography (HPLC). The cultivation experiment at 0.1 mM concentration was performed in replicate and only representative results are presented here.

#### Xenobiotic uptake

To confirm that the investigated compounds in the exposure solutions were taken up by plants and did not simply degrade, evaporate or adsorb to the beaker walls, another cultivation experiment was performed under similar conditions as described above. In this case, only 0.1 mM exposure was tested, which was also selected as optimal conditions for our ongoing studies. Freeze-dried root samples were analyzed by attenuated total reflection Fourier transform infrared spectroscopy (ATR FTIR, in detail described below). Moreover, root and shoot water extracts were prepared by mixing 50 mg homogenized lyophilized material with 2 ml Milli-Q water. After 48 h at 4 °C, the extracts were filtered through a 0.22 µm PTFE syringe filter and analyzed by HPLC (roots) and liquid chromatography mass spectroscopy (LC–MS; roots and shoots) for target xenobiotics. Details on the analyses are given in the Experimental methods section.

### Chemicals

Guaiacol and 4-nitroguaiacol (97%) standards were purchased from Sigma Aldrich.

### Experimental methods

#### Photochemical efficiency of photosystem II

The maximal photochemical efficiency of the photosystem II (Fv/Fm) was measured on the leaves with a modular fluorimeter (OS-500, Opti-Sciences, Hudson, NH, USA) at the start of exposure, after one week, and after two weeks of plants exposure. The Chlorophyll fluorescence was excited with a pulse of white light and the emitted light was detected in the 710–760 nm wavelength range. The measuring area was first adapted to dark conditions with plastic clips that were clamped onto the leaves. The adaptation to dark conditions was performed for 20 min prior to measurements.

#### Plant harvesting and preparation

At the end of a two-week exposure, the plants were harvested, washed with tap and distilled water, and separated into roots and shoots. The separate plant organs were weighed (fresh weight, FW) and frozen in liquid nitrogen. The material was freeze-dried for 3 days (0.001 mbar, − 95 °C, ScanVac, Labo-Gene, Denmark), weighed (dry weight, DW) and used for photosynthetic pigment content analysis. Water content (WC) was calculated as $\left( {FW - DW} \right)/FW$.

#### Photosynthetic pigments

Samples were prepared according to an adapted method by Monni et al*.*^41^. 30 mg of freeze-dried shoots were weighed into centrifuge tubes and 5 mL of 80% acetone were added. The samples were mixed by vortexing and incubated overnight at 4 °C. The next day, acetone was added to the final volume of 5 mL. Samples were again mixed by vortexing and centrifuged for 2 min at 2500 g. Absorbances of the supernatant were measured at 470, 647, and 664 nm with a UV-1800 spectrophotometer (Shimadzu Scientific Instruments, MD, USA) and sample pigment content was calculated according to Lichtenthaler and Buschmann^42^.

#### Chromatographic analysis

Chromatographic analyses of nutrient solutions and plant extracts were performed on an UltiMate 3000 UHPLC system (Thermo Scientific, Waltham, MA, USA) equipped with a diode-array detector and a triple quadrupole/linear ion trap mass spectrometer (4000 QTRAP LC–MS/MS System; Applied Biosystems/MDS Sciex, Ontario, Canada) with electrospray ionization (ESI). HPLC and LC–MS methods used are described in detail elsewhere^26,43^. Separation of target compounds was achieved on a reversed-phase analytical HPLC column. Milli-Q water (Millipore, Bedford, MA, USA) was used for the preparation of mobile phases.

In LC–MS analyses, formic acid (LC–MS grade) was used as a modifier. The screening Q1 experiments were performed in the *m/z* range of target compounds, whereas we never expected to see GUA as it does not ionize in ESI. Fragmentation patterns of perspective peaks with target *m/z* values were compared with the corresponding standards, which confirmed their identity (data not shown). An MS/MS experiment was further conducted to confirm the presence of anticipated nitrated components.

#### ATR FTIR analysis

Freeze-dried maize and sunflower roots were analyzed using a Tensor 27 infrared spectrometer equipped with an MCT detector and a diamond ATR cell (Golden Gate, Specac, Orpington, England). Typically, 64 scans acquired at room temperature were averaged and apodized using the Happ-Genzel function. The resolution of the recorded spectra is 2 cm^−1^. Differential spectroscopy was used to identify the presence of GUA and 4NG, i.e. the corresponding control spectra were subtracted from the spectra of the treated samples and the differential spectra were compared with the spectra of standard compounds.

### Statistical analysis

Standard deviation (SD) and standard error (SE) were calculated for every measurement parameter. Since the number of samples was low, normality of sample distributions was visually assessed using histograms and Kruskal Wallis nonparametric test was used to compare the effects of different exposures followed by multiple pairwise comparisons using Dunn's procedure (XLSTAT, Lumivero). Differences at *p* < *0.05* were considered significant. Statistically significant differences are denoted by letters, with each letter corresponding to a statistical group (e.g. groups “*a*” and “*b*” are statistically significantly different, while group “*ab*” is not statistically significantly different from “*a*” and/or “*b*”). A two-way clustering analysis (based on Euclidian distance) including all measured physiological parameters in all exposures as variables and a heat chart of measured parameters were performed in the “R” project for statistical computing (i386 3.4.3)^44^ on Z-transformed data^45^.

### Experimental research and field studies on plants statement

In the study only cultivated plants were used which are neither endangered nor at risk of extinction. We confirm that their handling was performed in compliance with relevant institution, national and international guidelines and legislation.

### Supplementary Information

Supplementary Figures.

## Data Availability

The datasets used and/or analyzed during the current study available from the corresponding author on reasonable request.
