# Supplementary material for: Toxicity of nitrophenolic pollutant 4-nitroguaiacol to terrestrial plants and comparison with its non-nitro analogue guaiacol (2-methoxyphenol)
Source: Sci Rep. 2024 Jan 25;14:2198. doi: 10.1038/s41598-024-52610-6 (PMC10811240; doi:10.1038/s41598-024-52610-6)
Supplement: Supplementary file 1 — Supplementary Figures. [file 41598_2024_52610_MOESM1_ESM.docx]

# Supplementary Information for

**Toxicity of nitrophenolic pollutant 4-nitroguaiacol to terrestrial plants and comparison with its non-nitro analogue guaiacol (2-methoxyphenol)**

Maksimiljan Adamek^a,b^, Anja Kavčič^c^, Marta Debeljak^a^, Martin Šala^a^, Jože Grdadolnik^d^, Katarina Vogel‑Mikuš^c,e^, Ana Kroflič^a,f,*^

^a^Department of Analytical Chemistry, National Institute of Chemistry, Hajdrihova 19, SI-1000 Ljubljana, Slovenia

^b^Department of Molecular Biology and Nanobiotechnology, National Institute of Chemistry, Hajdrihova 19, SI-1000 Ljubljana, Slovenia

^c^University of Ljubljana, Biotechnical Faculty, Department of Biology, Jamnikarjeva 101, SI-1000, Ljubljana, Slovenia

^d^Theory Department, National Institute of Chemistry, Hajdrihova 19, SI-1000 Ljubljana, Slovenia

^e^Jozef Stefan Institute, Jamova 39, SI-1000 Ljubljana, Slovenia

^f^Department of Catalysis and Chemical Reaction Engineering, National Institute of Chemistry, Hajdrihova 19, SI-1000 Ljubljana, Slovenia

A

B

Figure S1: Biomass (m) values for plant fresh weight (FW) and dry weight (DW) for A) maize and B) sunflower roots and shoots after two weeks of exposure to 0.1, and 1.0 mM GUA and 0.1, and 1.0 mM 4NG. In 1 mM 4NG exposure, roots were completely destroyed and could not be shown in this figure. Control plants (CON) are shown for comparison. Columns represent mean values (n=4), error bars are standard errors (SE). Letters above columns represent statistical groups following Kruskal-Wallis and Dunn’s post hoc test: different letters denote statistically significant differences (p<0.05).

| 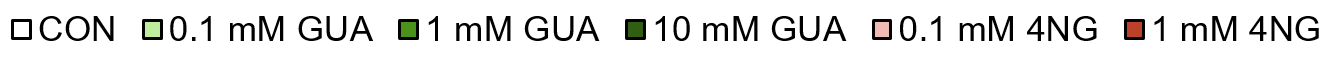 | |
| --- | --- |
| A | B |

Figure S2: Maximal photochemical efficiency (*Fv/Fm*) of maize (A) and sunflower (B) plants at the beginning of the experiment and after one and two weeks of exposure to 0.1, 1.0, and 10 mM GUA and 0.1, and 1.0 mM 4NG. Control plants (CON) are shown for comparison. Columns represent mean values (n=8), error bars are standard errors (SE). Letters above columns represent statistical groups following Kruskal-Wallis and Dunn’s post hoc test: different letters denote statistically significant differences (p<0.05).

A

B

Figure S3: Photosynthetic pigment concentrations in A) maize and B) sunflower plants after two-week exposure to 0.1, and 1.0 mM GUA and 0.1, and 1 mM 4NG expressed in mg of photosynthetic pigments per g of dry plant biomass. Control plants (CON) are shown for comparison. Columns represent mean values (n=4), error bars are standard errors (SE). Letters above columns represent statistical groups following Kruskal-Wallis and Dunn’s post hoc test: different letters denote statistically significant differences (p<0.05).

| A | B |
| --- | --- |
| C | 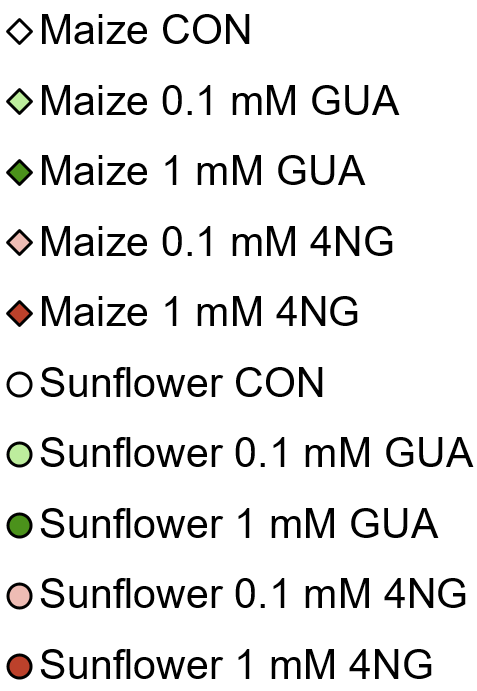 |

Figure S4: Maximal photochemical efficiency (*Fv/Fm*) after the two-week exposure of maize and sunflower plants to 0.1, and 1.0 mM GUA and 0.1, and 1.0 mM 4NG vs. photosynthetic pigment concentrations (expressed in mg of photosynthetic pigments per g of dry plant biomass) after two weeks of exposure: A) chlorophyll *a*, B) chlorophyll *b*, and C) carotenoids. Control plants (CON) are shown for comparison. Data points represent mean values of *Fv/Fm* (n=8) and sum of photosynthetic pigments (n=4), error bars are standard errors (SE).

Figure S5: ATR spectra of A) maize and B) sunflower roots together with the corresponding difference spectra between the treated and control samples. The red line represents the differences between the control samples, the black line represents the difference between the spectrum of the GUA treated maize roots and the control sample, and the blue line represents the difference between the spectrum of the maize roots treated with 4NG and the control sample.

Figure S6: Typical LC-MS TIC chromatograms of A) maize and B) sunflower root extracts for plant exposure to 0.1 mM 4NG compared to control samples. Only nitrated compounds are assigned. The peak at approximately 10 minutes includes both, 4NG and 4NS compounds, which were confirmed by MS/MS experiments.
